# Thirty years of great ape gestures

**DOI:** 10.1007/s10071-018-1167-1

**Published:** 2018-02-21

**Authors:** Michael Tomasello, Josep Call

**Affiliations:** 1 Max Planck Institute of Evolutionary Anthropology, Leipzig, Germany; 2 Department of Psychology and Neuroscience, Duke University, Durham, USA; 3 School of Psychology and Neuroscience, University of St Andrews, St Andrews, UK

**Keywords:** Apes, Gestures, Communication

## Abstract

We and our colleagues have been doing studies of great ape gestural communication for more than 30 years. Here we attempt to spell out what we have learned. Some aspects of the process have been reliably established by multiple researchers, for example, its intentional structure and its sensitivity to the attentional state of the recipient. Other aspects are more controversial. We argue here that it is a mistake to assimilate great ape gestures to the species-typical displays of other mammals by claiming that they are fixed action patterns, as there are many differences, including the use of attention-getters. It is also a mistake, we argue, to assimilate great ape gestures to human gestures by claiming that they are used referentially and declaratively in a human-like manner, as apes’ “pointing” gesture has many limitations and they do not gesture iconically. Great ape gestures constitute a unique form of primate communication with their own unique qualities.

There are two broad perspectives from which human languages may be viewed. First, they may be viewed as systems of representation in which symbolic vehicles represent external realities. This is the perspective of many cognitive scientists, especially those espousing the so-called representational theory of mind. Second, human languages may be viewed as conventionalized forms of social action in which communicative agents attempt to influence one another’s psychological states in various ways. This is the perspective of an otherwise diverse group of social scientists from various disciplines, who accept that a language is a representational medium but at the same time insist that the representational function operates always and only in the service of the social function. Does that colorful piece of cloth represent a country (or does that funny sound represent X)? Only if someone intends that someone else take it to do so.

The great discovery of Seyfarth et al. (1980) was that some forms of primate vocal communication (e.g., vervet monkey alarm calls) seem to operate representationally: Different calls are used systematically in association with specific classes of referents. The implications of this discovery for the evolution of human linguistic communication were immediately obvious. But further research soon documented limitations in individuals’ ability to use such representational vocalizations to communicate flexibly with different recipients, in various psychological states, in a range of social circumstances (see, e.g., Cheney and Seyfarth 1990). Indeed, the caller seemed to have very little intentional control over the production of the vocalization at all: “Listeners acquire information from signalers who do not, in the human sense, intend to provide it.” (Seyfarth and Cheney 2003, p. 168); “Nonhuman primates vocalize in response to important events, irrespective of how potential recipients may view the situation.” (Zuberbühler 2005, p. 126, but see Schel et al. 2013; Crockford et al. 2017). In addition, it was perhaps noteworthy that great apes, as humans’ closest primate relatives, did not seem to have these same kinds of referentially specific calls, at least not to the same extent as various species of monkeys (and prairie dogs and chickens).

It is in this context, in the 1980s, that we began our studies of great ape gestural communication. The focus from the beginning was on gestures as social action aimed at influencing others, taking as a starting point the many insightful observations of field ethologists on the communicative actions and displays of species ranging from fish to birds to dogs. Specifically, our initial study was prompted by two ethological papers in the volume *Action, Gesture, and Symbol: Emergence of Language* (Lock 1978). In one of these, Plooij (1978) reported and discussed an ethogram of chimpanzee gestures, based on observations of the infants and juveniles in the Gombe (Kasakela) community. What was remarkable was that these gestures did not seem to be the kind of fixed action patterns characteristic of the phylogenetically ritualized communicative signals of the ethologists (e.g., bird mating displays). They seemed to be much more under the intentional control of the individual for flexible use as needed in particular social circumstances. In the other paper, Locke (1978) reported some ethological observations of human infants requesting to be picked up, most often using the well-known gesture of “arms up” toward the adult. This gesture was not like the most studied gestures in child development, namely pointing and the use of iconic signs, and it certainly was not conventional linguistic communication. Instead, it seemed to be a kind of learned “intention-movement” (Tinbergen 1951, 1952): The baby was using a reaching-up movement not to actually crawl up into the adult’s arms but rather to prompt the adult to effect that end for her. On the surface, at least, there seemed to be a remarkable similarity to some of the chimpanzee gestures Plooij was reporting.

And so we went to watch the Yerkes chimpanzees. We went armed with three basic questions: (1) Did chimpanzees use at least some of their gestures flexibly and under their own intentional control?; (2) how did they learn their gestures, if indeed they learned them at all?; and (3) how did they understand what they were doing (i.e., what underlying processes of social cognition were involved)? In the background was perhaps the somewhat larger question of how chimpanzees’ gestural communication related to the evolution and development of human language. What ensued was a series of four observational studies (Tomasello et al. 1985, 1989, 1994, 1997), carried out on two groups of captive chimpanzees at the Yerkes Primate Center, comprising one to two dozen individuals each. Later, after we arrived at the Max Planck Institute in Leipzig in 1998, we and our colleagues (especially Katja Liebal and Simone Pika) expanded both the ape species studied and the questions asked. We summarized much of this work in an edited volume (Call and Tomasello 2007) and further developed some theoretical implications in a book on the evolution of human communication (Tomasello 2008).

In the past decade or so a number of other researchers have also begun to investigate great ape gestural communication, including importantly in its natural habitats in the wild. Many of their observations and interpretations are broadly consistent with ours, but some disagreements have arisen as well. Our goal in this paper, after a brief account of the most important areas of agreement, is to address the most important of the outstanding disagreements. In general, we will defend the view that much (not all) of great ape gestural communication is intentional and learned, but at the same time it differs from human gestural and linguistic communication in being fundamentally individualistic rather than cooperative. Great ape gestural communication is a sophisticated form of individual intentionality, not human-like shared intentionality (Tomasello 2014).

## Ape gestures as intentional communication

First the basic agreements. From the beginning, what made great ape gestures stand out from the communicative displays of many other species was the flexibility, and seeming deliberateness, with which they were used. Following Bruner’s (1981) operational definition of intentional action, we singled out from chimpanzees’ social interactions specific acts that individuals used repeatedly toward a recipient until they got a particular response, at which point they ceased acting. Moreover, there was not a simple one-to-one mapping between such acts and their functions across instances (as is typical with fixed displays); one act might be used for different functions in different contexts, and one function might be effected by different acts. We called these acts “signals” because, following Smith (1977), the communicator was not attempting to affect the recipient physically, but rather psychologically; she got the other to do what she wanted not by forcing him physically, but rather by displaying her intended social action and waiting for a reaction.

Bolstering this finding that chimpanzees use their gestural signals intentionally, with the aim of affecting the psychological states of others, are two other findings. First, with respect to the intentional dimension, is the finding that chimpanzees often produce sequences of gestures toward a specific recipient seemingly in pursuit of a single social goal, for example, they might use a string of multiple different play gestures until the other begins playing (Liebal et al. 2004a). Hobaiter and Byrne (2011b) analyzed chimpanzee gesture sequences and found something very similar, concluding that individuals were producing different individual gestures until one of them worked. Persistence to a goal, trying alternate means as necessary, is one of the hallmarks of intentional action. And the fact that they are aimed at other individuals (whereas vocalizations are mostly broadcast indiscriminately in an area) suggests that the goal was indeed a social (communicative) goal. Similar results have been found with orangutans (Cartmill and Byrne 2010).

Second, with respect to the psychological dimension, we also discovered more powerful audience effects in chimpanzee gestural communication than those reported in monkey vocal communication. Whereas vervet monkeys were more likely to use a vocalization when specific others (e.g., kin) were present versus absent, we found that the apes took into account the perceptual access of the recipient to the signal. Specifically, visual signals that required that the recipient sees them if they were to work were given most often when the recipient was actually looking—whereas this was not true of tactile signals (Tomasello et al. 1994). They also use their attention-getting gestures (see below) to get others to look at them when that would lead to the desired result (e.g., they already had an involuntary play face, but the other was not looking at it). Moreover, on some occasions if the recipient was not looking, the communicator would move around in front of her to make sure she saw the signal (Liebal et al. 2004a). We also documented such audience affects experimentally, finding that apes do indeed walk around as necessary to signal visually to the face of the recipient (Liebal et al. 2004b). These observations have been replicated by many different investigators, most prominently, Genty et al. (2009) and Hobaiter and Byrne (2011a). Appreciation that a signal has to be seen to be responded to, again, suggests a process aimed at the psychological states of others.

Great ape gestural communication interpreted as intentional social acts aimed at influencing the psychological states of others is thus a generally accepted conceptual framework for the study of one important form of primate communication. (Although we should note that getting our first study published in an animal behavior journal in the mid-1980s proved to be impossible because behaviorist reviewers continually objected to calling these behaviors “intentional.”) Our current best attempt to operationalize an intentionally produced gesture is: “a behaviour that unlike an action is motorically ineffective. It requires the active participation of a partner to fulfil its purpose, it is produced in the presence of an audience and is tailored to the attentional state of the audience. Furthermore, it involves gaze alternation or visual checking between social partners and distant objects or events, is characterized by the sender’s waiting for the recipient’s response and displays persistence and elaboration of communicative behaviour when communicative attempts fail”. (Liebal and Call 2012, p. 119, although the gaze alternation to distant events is important in only some instances).

## Ontogenetic ritualization and the distinction between intention-movements and attention-getters

Now to some disagreements. Beginning with Tomasello et al. (1989), we systematically distinguished between two types of great ape gesture. From our first observations we had been focused on the kinds of *intention*-*movements* that ethologists had been observing for several decades, though in our case we thought they were not phylogenetically ritualized but rather individually ritualized. These were things like an ape infant who, instead of grabbing the hair on mom’s back and pulling herself up, simply touched mom lightly on the back inducing her to lower her back to enable climbing on. Or a youngster raised its arm toward another hesitantly, as if about to hit the other one over the head, to initiate play. In both cases the actual act, the gestural signal, appeared to be “ritualized” in the sense that it was not adequate to force its desired outcome physically, but rather it was a truncated version of a naturally meaningful social behavior. The “meaning” of the gesture derived from the meaning of the original social act.

But some gestures did not fit this pattern. For example, to initiate play juveniles quite often would slap the ground noisily toward another, or throw something at another, or poke another in the back vigorously. The signaler clearly was attempting to induce play (he had a play face), but these were not intention-movements of normal play behaviors. They seemed rather to be aimed at getting the other to look in the direction of the noise-maker or the thrower or the poker. The signaler’s desire to play was expressed in the species-typical display of a play face and posture. So in this case the process was a bit more indirect. The signaler seemingly took control of the process by which her species-typical displays were perceived and responded to by a recipient. They were thus somewhat analogous to reports of great apes doing such things as hiding a fear grimace or play face with their hands (e.g., Tanner and Byrne 1993), or male chimpanzees in the wild stripping dried leaves from their stem loudly in order to get females’ attention to their sexual arousal (leaf-clipping; Nishida 1980). In this case, the “meaning” of the gesture derived from the species-typical display, and the *attention*-*getters*, as we called these signals, were aimed at getting the recipient to attend to the display so that she could respond to it.

We wondered about the learning process for these two types of signal. We first focused on how intention-movements were learned, and indeed, we assumed that they were learned because they were not like species-typical displays, which are most often fairly stereotyped, inflexible, and associated with only a single function. Our main strategy was to investigate the possibility that these gestures were learned through imitation, and we concluded that they were not. Tomasello et al. (1994) looked at similarities and differences between two groups of captive chimpanzees and found as much variability within groups as between groups. In addition, in this same paper we reported an experimental study in which we trained an individual in a gesture and put her back in the group to see whether anyone would imitate it. No one did. Although there are some suggestive observations in several different studies, in general researchers such as Genty et al. (2009) and Hobaiter and Byrne (2011a) have confirmed that imitation does not play a major role in great apes’ acquisition of gestures. So, we assumed that the process was very likely one that paralleled the ethologists’ notion of ritualization, but in this case the ritualization was not accomplished through natural selection but through learning.

In our earliest descriptions, we were not very clear about how much the process was a more low-level mutual shaping or a more cognitive one in which individuals understood what the other was intending to do (And indeed at the time, based on available evidence, we tended toward the leaner interpretation). But the way we would describe it now is that an individual actually performs some social act toward a recipient, and over repeated instances the recipient starts anticipating what the actor intends (or will do) based on some initial part of the act; the actor notes that the recipient anticipates his intention (or what he will do) on the basis of this initial sequence, and infers a causal link in the sense that he understands that it is this initial part of the act that instigates the reaction. The initial part of the act becomes “emancipated” from the physically efficacious dimensions of the original social act; it becomes ritualized. We observed many gestures that seem to have this form, like the touchback for infants wanting to climb on mom’s back and the arm-raise for initiating play with peers cited above. But the attention-getters could not be learned in exactly this way. So we hypothesized that individuals did things that made noise for whatever reason, and then observed that when they did so others tended to look in their direction. But we did not pursue this hypothesis further to try to nail down the acquisition process of attention-getters in more detail, beyond documenting (Tomasello et al. 1997) that the precise actions involved varied quite widely even for the same individual on different occasions (e.g., ground-slap covered all kinds of slapping actions on all kinds of substrates), suggesting not an inflexible species-typical display, but rather an action defined by its function, in this case the social function of drawing attention to the self.

In a series of papers over the past few years, Byrne and colleagues have argued for a different account of great ape gestures (see Byrne, this volume). They believe that great ape gestures are basically just species-typical communicative displays that have somehow come under flexible intentional control. In this sense, gestures are not so different from vocalizations whose mostly inflexible nature in production is amply demonstrated (e.g., Hammerschmidt and Fischer 2008). In arguing for this account, they have criticized our account on two basic points. First and most directly, they have questioned whether great apes actually learn gestures through ontogenetic ritualization. In a curious analysis Hobaiter and Byrne (2011a) chose two chimpanzee gestures that resemble a social action used for the same function: the begging-reach gesture, potentially ritualized from actually taking food, and the position gesture, potentially ritualized from attempting to physically position a grooming partner. In both cases the gesture clearly resembles the social action: “these gestures and actions were … similar: they were, after all, originally picked out as having… basic similarity of form” (Hobaiter and Byrne 2011a, p. 763). But they then proceed to analyze the actual body movements in minute detail for such things as: the orientation of the palm, the position of the fingers, and the part of the hand that was presented first. Because at this level of detail the ritualized gesture was different from the social action, they conclude that the gesture was not ritualized from the action. But, of course, one could also argue that such things as the orientation of the palm with the position of the fingers are not the level of detail at which the chimpanzees understand and produce the social action. (And it is likely that the begging-reach gesture is not ritualized from taking food but from holding the hand under the mouth of the eater, which accounts for the palm oriented up.)

In response, Halina et al. (2013) studied in detail the gestures used by 10 bonobo infants and their mothers to initiate carries, that is, the mother carrying the infant on the back in travel (see Rossano, this volume, for more detail). Importantly in the current context, the social actions involved in initiating this behavior are different when they come from the mother (who most often grasps the infant and places it on her back) and from the infant (who most often attempts, in one way or another, to climb on), providing different raw material for the ritualization process in the two cases. A main finding was that there were four gestures used only by infants, two gestures used only by mothers, and three gestures that were used by both, albeit in very different ways. In each case the gesture used resembled a corresponding social act. For example, the spread-legs gesture was used by three of the infants to request a carry: The infant would hang from a rope or branch by her hands and reach out toward the mother with her legs. In the social action, the infant did this when the mom was right below her and she could just climb on, but when it was used gesturally, the infant used a truncated version from a distance to request that the mother come over to her so she could climb on. Importantly, the three infants that used this gesture came from the same social group, whereas the seven other infants did not use it at all. This was mainly because the three gesturing infants lived in a physical setting with many opportunities for hanging by the hands, whereas the others did not. There was also one idiosyncratic gesture used by only one infant (spin body), and not by others, even those who were observed for many hours (up to an asymptote such that additional hours observation revealed no new gestures). We would argue that these observations establish quite solidly that at least some great ape gestures are ontogenetically ritualized.

Byrne and colleagues also question the distinction between intention-movements and attention-getters. This is based partly on their doubting that some ape gestures are actually ontogenetically ritualized social actions, as just detailed. Genty et al. (2009) found for gorillas that gestures that resembled corresponding social actions to some degree and gestures that did not were both used equally flexibly and for similar functions (e.g., for chase, cuddle, etc.). But the key point is that even if they are both used flexibly for the same final function, for example, play, they work in different ways. For instance, slapping the ground or throwing stuff at a partner, as attention-getters, does not directly relate to play in a way that the recipient could be expected to understand, and indeed in our original studies both the ground-slap and throw-stuff gestures were used in other contexts as well, for example, requesting that mom allow nursing. Instead, ground-slap and throw-stuff as requests for play were understood as such by the recipient because the gesturer was in a play posture with a play face, and it is these involuntary species-typical displays that actually convey the content of the communicative intention. (The desire to nurse, in the other context, was expressed through something like repeated (and rebuked) attempts to access the nipple paired with a pout-face display.) It is also worth mentioning that great apes often learn many novel attention-getting behaviors when interacting with humans including clapping their hands, pointing, and spitting, in ways that are not frequently observed in other primate species. The large potential for gestural learning is fully revealed by apes acquiring rudiments of a human sign language (e.g., Gardner et al. 1989).

We believe that the distinction between intention-movements and attention-getters is fundamental because, again, they work in different ways: either directly on the recipient’s psychological states, in the case of intention-movements, or indirectly on those same states via attention manipulation, in the case of attention-getters. Indeed, we would argue that even in the case of clearly inflexible species-typical displays, such as the gorilla chest-beat, one may specify whether the gesture is attaining its goal more directly or more indirectly through attention manipulation. So we would classify gorilla chest-beat is an attention-getter (typically to displays of dominance/aggression), even if it is unlearned. Importantly, as Tomasello (2008) argues, the distinction between intention-movements and attention-getters can even be seen in humans’ two most basic types of natural gestures (i.e., excluding conventional gestures or those relating to language): pointing, which aims at manipulating another’s attention (and so are like attention-getters), and pantomime, which prototypically represents meaningful social acts symbolically (and so are elaborated symbolic versions of intention-movements). This potential connection to the human case makes the distinction between intention-movements and attention-getters even more plausible and even more crucial. It is nevertheless true that we do not know how specific attention-getters are acquired—if indeed they are acquired—in individual cases. Their acquisition has not been studied in the kind of detail that intention-movements have been (as in, e.g., Halina et al. 2013).

To summarize, clearly much great ape communication occurs via relatively inflexible species-typical displays, as in the many other species studied by ethologists. Many of these were originally the initiating action of a meaningful social act, which then became phylogenetically ritualized, as the ethologists have documented. How could it be otherwise? Gestures must get their “meaning” from somewhere, and inherently meaningful social actions are pretty much the only candidate. And so the question is, if the Byrne et al. account has some validity, how did apes seemingly get more flexible intentional control over their species-typical displays? One possibility is through a variant on ontogenetic ritualization. That is to say, intelligent ape individuals see the reaction of others to their involuntary displays, and they learn a causal connection, which enables them to now produce it flexibly and cognitively as needed in appropriate circumstances. (Note that Tinbergen himself (1952, p. 1) questioned the notion that, at least in the case of birds, individuals may recognize this causal connection.) In addition, however, we would argue that great apes also learn at least some gestures in interaction with humans (e.g., Gardner et al. 1989; Gomez 1990) and conspecific as indicated by developmental data (e.g., Halina et al. 2013) and the existence of idiosyncratic gestures (Call and Tomasello 2007). What proportion that might be is not known, and indeed, it might even differ for the different great ape species (with gorillas doing less learning). Or it might even differ for different individuals within a species. Halina et al. (2013) speculate in this case that when individuals face uncooperative partners, who do not respond as desired to social actions, they might face special social pressure to gesture and to produce it insistently.

## Ape gestures as mainly dyadic and imperative

A second set of disagreements comes from the other direction, as it were, that is, from those who see great ape gestures as more human-like (e.g., Leavens et al. 2015; Lyn et al. 2010). They see at least some great ape gestures as fully referential and declarative, that is, not just imperative (see below).

From our very first studies we stressed that almost all of the great ape gestures that we observed were dyadic in the sense that they serve to regulate a direct social interaction, for example, playing, grooming, carrying, and so forth, with no external referents involved. Interestingly, however, attention-getters do direct attention to the self, or some body part in some cases, which is not a third object but still involves something in the direction of reference. In addition, we and others have noted a few gestures that have something of a third element in them, as when one individual in a play mood holds out an object to another and quickly retracts it as an inducement to play, and we and others have also noted some food-begging gestures. In Tomasello et al. (1985), we even reported on an individual who used a gesture we call “point,” originally with humans, to indicate to its mom where on his body he wanted to be tickled, and something similar has also been observed in the wild in the context of grooming (so-called directed scratch, Pika and Mitani 2006). Hobaiter et al. (2013) reported four observations of chimpanzees from the wild (three involving a single mother–child pair on a single occasion) in which a juvenile extended its hand and arm toward “a desirable but unobtainable object.” (Vea and Sabater-Pi 1998 also report a potential case of bonobo pointing in the wild with an unclear function.)

So clearly great apes know something about directing the attention of the other. But is their understanding such that we should attribute to them a human-like understanding of the act of reference? Are their gestures fully referential in both production and comprehension? To determine this we need experiments, and these mostly involve apes communicating with humans. The most systematic work has been done by Leavens et al. (e.g., Leavens and Hopkins 1998; Leavens et al. 2005), and they have found that chimpanzees will point for humans with persistence and gaze alternation between a human and a desired object. Bohn et al. (2015, 2016a; see also Lyn et al. 2014) even found that apes will point to an empty plate to request food of the type that used to be on the plate previously. They are directing attention in flexible ways so that others will see things that make them react in desired ways. However, in experiments in which they must comprehend human pointing gesture, great apes have surprising difficulties. Many different studies have found that great apes fail to comprehend a human’s communicative intention when he points to the bucket (out of two or three) that contains food the ape desires (see Tomasello 2006, for a review; see Mulcahy and Call 2009, for an exceptional result). This comprehension failure might explain why apes point for humans but not for one another; why point for someone who does not respond appropriately?

One interpretation of great ape pointing is that it is a kind of ritualized reaching that humans (but not other apes) respond to as if it were efforts at real reaching, by retrieving the object for them. Apes themselves do not comprehend the pointing gesture as referring them helpfully to the location of desired food, but instead they interpret it as a ritualized reach for the pointer’s own benefit (which they have no desire to fulfill). Two other experiments also help us to interpret the nature of great ape pointing. First, we should note that great apes do not point in any studies by extending their index finger toward distant objects, but rather they extend their whole hand to close-by objects, most often resting their hand in the mesh of caging or the like. Starting with this observation, van der Goot et al. (2014) presented chimpanzees with a desirable object next to a human but some distance away. Chimpanzees basically never pointed to the desired object, but instead locomoted over to it and then reached ritualistically through the mesh for it (i.e., “pointed” for the human close to it). Human infants in the same situation mostly just pointed from a distance. Second, Halina et al. (in press) had a human respond to apes’ pointing to food either by looking at it but not responding (unwilling condition) or by looking in a wrong direction (misunderstanding condition). Apes did not respond differently in the two different conditions, suggesting that they do not distinguish whether the recipient is unwilling to accede to their desire or is unable to comprehend the reference of the pointing; they just know it did not work. A reasonable overall interpretation, therefore, is that chimpanzees’ pointing comprises commands to humans effected via ritualized reaching, and their comprehension is poor because they understand others not to be informing them of something helpfully but to be reaching ritualistically for themselves.

This interpretation is bolstered by the fact that great ape pointing—as well as all other forms of their gesturing—is almost always imperative in function. That is, apes do not point declaratively to simply share interest and attention in something with another individual, and they do not point to inform others of things they might want or need to know—as human infants do from very early in ontogeny. Tomasello and Carpenter (2005) presented three young human-raised chimpanzees with situations that reliably elicit such declarative pointing in human infants (e.g., surprising, interesting events), but observed no declaratives from them in response. Tomonaga et al. (2004) also report no declarative pointing or gesturing in their young chimpanzees despite many situations designed to elicit them. And even the signed productions of “linguistic” apes are almost all imperatives—approximately 96–98% in the only two systematic studies (Rivas 2005; Greenfield and Savage-Rumbaugh 1990), with the other 2–4% having no clear functional interpretation. (They are not clearly declarative or informative, but more recognitory or classificatory, as the ape simply recognizes a stimulus and produces the associated sign in recognition.) Melis et al. (2009; see also Bullinger et al. 2011) report that even when informative gestures would be extremely helpful to them—helping their partner in a mutualistic collaborative context—apes do not produce them (even though they will help their partner physically in the same situation; Melis and Tomasello 2013). This functional restriction to imperative gesturing also contributes to apes’ surprising troubles in comprehending human pointing gestures designed to inform them of things.

Finally, we should also note the great apes do not seem to produce or comprehend iconic gestures, that is, symbolic depictions intended to indicate external referents, for example, miming eating to request food, or making a hammering motion to request a stone hammer. Some researchers have claimed that some intention-movements are actually functioning iconically, for example, when one gorilla ritualistically motions another in a direction in a sexual or play context (Tanner and Byrne 1996). But these are most likely garden-variety ritualized behaviors that appear to humans to be iconic because they derive from attempts to actually move the body of the other in the desired direction, but they are not functioning iconically for the apes themselves. In experiments, apes have systematically failed to either produce or comprehend iconic gestures in situations where it would benefit them to do so. For example, Grosse et al. (2015) set up a situation in which an ignorant human needed to know how to operate an apparatus to retrieve food for the ape, but they did not show her how to do it, even though they knew how to do it themselves. Bohn et al. (2016b) gave apes an iconic gesture showing them which apparatus they should operate in order to get food (i.e., the one that works like “this”), but again to no avail. It is not likely that the apes fail to see any resemblance between the iconic gesture and some referent (see Buttelmann et al. 2013), but more likely is that they do not understand the declarative referential act as such.

Tomasello (2008) concludes from these and other considerations that although great apes do understand about directing the attention of others to things, they do not understand reference in human-like ways because human reference is, in effect, an invitation to share attention (Tomasello 1998). That is to say, a human act of reference may be glossed as: I am attending to something that I think you will find interesting, and I would like you to join me in attending to it (so that you will do something in response). Human communication is therefore deeply cooperative, based on cooperative motives and structured by individuals jointly attending to things of mutual interest. The deepest underlying issue that differentiates great ape gestural communication from human gestural communication, therefore, is cooperation. Great apes are essentially communicating in order to fulfill individualistic goals—and they understand others to be doing this as well—whereas humans are communicating cooperatively in the context of joint goals and joint attention.

## Ape gestural communication and the origins of human language

Our claim here, then, is that great ape gestural communication is its own unique system with its own unique qualities. Researchers such as Byrne and colleagues are essentially arguing that ape gestures are very similar to the communicative displays of other mammals (they have even likened them to some bird displays), whereas researchers such as Leavens and colleagues are arguing that they are very similar to human cooperative communication. Of course there are similarities in both directions, but there are unique qualities as well.

And so the study of great ape gestural communication is interesting and important in its own right as a unique system of animal communication. But it is also interesting and important because it very likely represents the evolutionary starting point for human linguistic communication. Human linguistic communication is a form of intentional social action, and as such, its most likely precursor in primate communication is not inflexible forms of vocal communication but rather intentionally and flexibly used gestural communication. Evidence for this proposal also comes from the fact that when deaf humans come together, they find it quite natural to create a conventional sign language in the gestural modality (e.g., Senghas et al. 2004; Sandler et al. 2005). Evolutionarily, it is most likely that early humans were using attention-getters and intention-movements in the gestural modality—at some point transformed into pointing and pantomiming—for some time before their vocalizations came under intentional control. Initially, they added voluntarily controlled emotional expression to the communicative act, and later the vocal modality became predominant for well-known reasons involving such things as the requirement for long-distance communication in conditions of poor visibility, the freeing of the hands for other activities, and so forth (Tomasello 2008).

In this context, we would reiterate the importance of the distinction between attention-getters and intention-movements as they represent precursors of the two basic ways that humans manipulate the attention and imagination of others gesturally: by pointing or otherwise deictically indicating something in the immediate perceptual environment and by pantomiming or otherwise symbolically representing something in the imagination. Scaling up to language, attention-getters represent a kind of “missing link” on the way to human reference because they communicate indirectly, via the manipulation of the attention of the recipient to specific entities, and intention-movements represent a kind of “missing link” on the way to conventional linguistic symbols because they are actions that evoke in the recipient imagined (but not yet present) events and objects (Tomasello 2008).

What is needed to get from great ape intention-movements and attention-getters to human linguistic communication is a transformation in human social life, specifically, one that leads to a more cooperative lifestyle, underpinned by skills and motivations for shared intentionality (Tomasello 2014). In this process, newly cooperative communicative motives emerge (i.e., declarative and informative) and fully developed processes of reference—as an invitation to joint attention—and conventional symbolic representation—as a socially shared medium of expression—transform the nature of communication and the cognitive processes underlying it. The study of great ape gestural communication, to repeat, is thus not only important and interesting in its own right, but it is also crucial to our understanding of the origins and evolution of human linguistic communication.
